# Safety and efficacy of nivolumab in combination with sunitinib or pazopanib in advanced or metastatic renal cell carcinoma: the CheckMate 016 study

**DOI:** 10.1186/s40425-018-0420-0

**Published:** 2018-10-22

**Authors:** Asim Amin, Elizabeth R Plimack, Marc S Ernstoff, Lionel D Lewis, Todd M Bauer, David F McDermott, Michael Carducci, Christian Kollmannsberger, Brian I Rini, Daniel Y C Heng, Jennifer Knox, Martin H Voss, Jennifer Spratlin, Elmer Berghorn, Lingfeng Yang, Hans J Hammers

**Affiliations:** 1 Immunotherapy program, Levine Cancer Institute, Carolinas HealthCare System, 1024 Morehead Medical Drive, Charlotte, NC 28204 USA; 2 Division of Genitourinary Medical Oncology, Department of Hematology/Oncology, Fox Chase Cancer Center, Philadelphia, PA 19111 USA; 3 Division of Oncology, Department of Medicine, Roswell Park Cancer Institute, Elm and Carlton Streets, Buffalo, NY 14203 USA; 4 Department of Medicine at The Geisel School of Medicine and The Norris Cotton Cancer Center at Dartmouth-Hitchcock Medical Center, Lebanon, NH 03756 USA; 5 Sarah Cannon Research Institute/Tennessee Oncology, PLLC, Nashville, TN 37203 USA; 6 Department of Medicine, Beth Israel Deaconess Medical Center, Dana-Farber/Harvard Cancer Center, Boston, MA 02215 USA; 7 Department of Oncology, Johns Hopkins Sidney Kimmel Comprehensive Cancer Center, Baltimore, MD 21287 USA; 8 Division of Medical Oncology, British Columbia Cancer Agency, Vancouver, BC V5Z 4E6 Canada; 9 Lerner College of Medicine, Department of Hematology and Oncology, Cleveland Clinic Taussig Cancer Institute, Cleveland, OH 44195 USA; 10 Department of Oncology, Tom Baker Cancer Center, University of Calgary, Calgary, AB T2N 4N2 Canada; 11 Cancer Clinical Research Unit (CCRU), Princess Margaret Cancer Centre, Toronto, ON M5G 1Z5 Canada; 12 Department of Medicine, Memorial Sloan Kettering Cancer Center, New York, NY 10065 USA; 13 Department of Oncology, Cross Cancer Institute, University of Alberta, Edmonton, AB T6G 1Z2 Canada; 14 Oncology - Global Clinical Research, Bristol-Myers Squibb, Princeton, NJ 08541 USA; 15 Department of Internal Medicine, UT Southwestern – Kidney Cancer Program, Dallas, TX 75390 USA

**Keywords:** Metastatic renal cell carcinoma, Nivolumab, Immune checkpoint inhibitor, Sunitinib, Pazopanib, Antiangiogenic, Tyrosine kinase inhibitor

## Abstract

**Background:**

Combination treatment with immune checkpoint inhibitors and antiangiogenic drugs has shown encouraging preliminary antitumor activity across various tumor types including advanced or metastatic renal cell carcinoma (aRCC). The open-label, parallel-cohort, dose-escalation, phase I CheckMate 016 study evaluated the efficacy and safety of nivolumab in combination with antiangiogenic tyrosine kinase inhibitors or ipilimumab. Long-term outcomes from this study for the combination of nivolumab plus sunitinib or pazopanib in aRCC are presented.

**Methods:**

Patients with aRCC received nivolumab plus either sunitinib (50 mg/day, 4 weeks on/2 weeks off; N + S) or pazopanib (800 mg/day; N + P) until progression/unacceptable toxicity. The nivolumab starting dose was 2 mg/kg every 3 weeks, with planned escalation to 5 mg/kg every 3 weeks. Primary endpoints were safety and tolerability; antitumor activity was a secondary endpoint.

**Results:**

Arm N + S enrolled 33 patients, 19 of whom were treatment-naïve; this arm advanced to the expansion phase. Median follow-up was 50.0 months. Patients experienced high frequencies of adverse events (AEs) including treatment-related AEs (100%), grade 3/4 treatment-related AEs (82%), and treatment-related AEs leading to discontinuation (39%). Investigator-assessed objective response rate (ORR) was 55% (18/33) and median progression-free survival (PFS) was 12.7 months. Median overall survival (OS) was not reached.

Arm N + P enrolled 20 patients, all had ≥1 prior systemic therapy; this arm was closed due to dose-limiting toxicities and did not proceed to expansion. Median follow-up was 27.1 months. Patients treated with N + P experienced high frequencies of AEs including treatment-related AEs (100%), grade 3/4 treatment-related AEs (70%), and treatment-related AEs leading to discontinuation (25%). Investigator-assessed ORR was 45% (9/20) and median PFS was 7.2 months. Median OS was 27.9 months.

**Conclusions:**

The addition of standard doses of sunitinib or pazopanib to nivolumab resulted in a high incidence of high-grade toxicities limiting future development of either combination regimen. While there was no adverse impact on response and the OS outcome was notable, the findings suggest that the success of combination regimens based on immune checkpoint inhibitors and antiangiogenic drugs may be dependent on careful selection of the antiangiogenic component and dose.

**Trial registration:**

Clinicaltrials.gov identifier: NCT01472081. Registered 16 November 2011.

**Electronic supplementary material:**

The online version of this article (10.1186/s40425-018-0420-0) contains supplementary material, which is available to authorized users.

## Background

Immunotherapeutic and antiangiogenic agents have improved treatment outcomes for patients with advanced or metastatic renal cell carcinoma (aRCC) [1–12]. Vascular endothelial growth factor (VEGF) receptor tyrosine kinase inhibitors (TKIs) are approved for first-and/or second-line aRCC treatment [3–5, 8–11]. Newer therapies targeting immune checkpoint pathways have also demonstrated significant clinical efficacy in aRCC, and are approved for this indication [2, 6, 7, 13].

The TKI sunitinib became a standard monotherapy option for treatment-naïve patients with aRCC after demonstrating superiority over interferon alpha; sunitinib has also demonstrated efficacy in pretreated patients [14]. In updated results from the key phase III trial comparing sunitinib with interferon alpha (*N* = 750), treatment-naïve patients with aRCC achieved an objective response rate (ORR) of 47% versus 12% (*P* < 0.001), a median progression-free survival (PFS) of 11 versus 5 months (*P* < 0.001), and a median overall survival (OS) of 26.4 versus 21.8 months (*P* = 0.051) [9]. In second-line trials of sunitinib in aRCC (post VEGF-targeted therapy), reported ORRs have ranged from 15 to 27%, and median PFS has ranged from ~ 5–18 months [14]. The TKI pazopanib has also demonstrated efficacy in treating first- and second-line aRCC [15]. In a phase III study (VEG105192) of pazopanib versus placebo in treatment-naïve or pretreated patients (*N* = 435), median PFS was 9.2 versus 4.2 months (*P* < 0.0001), and ORR was 30% versus 3% (*P* < 0.001) [11]. The median OS was 22.9 versus 20.5 months (one-sided *P* = 0.224), however, this analysis was confounded by the early, high rate of crossover of placebo patients to pazopanib [12]. In the open-label COMPARZ trial, which compared the efficacy and safety of pazopanib versus sunitinib as first-line therapy in 1110 patients with clear cell aRCC, PFS was 8.4 versus 9.5 months, respectively [8]. In an updated report, OS was found to be similar in both the pazopanib and sunitinib groups (28.3 vs 29.1 months) [16]. Sunitinib and pazopanib are considered to be similarly efficacious as first-line therapy in aRCC [17], and do not differentially impact outcomes with subsequent second-line treatment [18].

In a large expanded access program study (*N* = 4543), 95% of patients treated with sunitinib reported adverse events (AEs). The most commonly reported treatment-related grade 3 or 4 AEs included thrombocytopenia (10%), fatigue (9%), asthenia, hand–foot syndrome, and neutropenia (each 7%), hypertension (6%), and diarrhea (5%) [19]. In the VEG105192 trial, all patients in the pazopanib arm (*n* = 290) experienced ≥1 AE. The most common treatment-emergent grade 3 or 4 AEs with pazopanib were increased alanine aminotransferase (ALT; 12%), increased aspartate aminotransferase (AST; 8%), hyponatremia (5%), lymphocytopenia (5%), hypophosphatemia (4%), hypertension (4%), and diarrhea (4%) [11].

Nivolumab, a fully human IgG4 programmed death-1 (PD-1) immune checkpoint inhibitor antibody, selectively blocks interaction between PD-1 expressed on activated T cells and the PD-1 ligands 1 and 2 expressed on tumor cells and other immune cells [20]. This blockade of PD-1–mediated signaling prevents T-cell inactivation and enhances antitumor immunity [20]. In the CheckMate 025 randomized phase III study (*N* = 821), median OS was longer with nivolumab compared with everolimus (25.0 vs 19.6 months; *P* = 0.002) [7]. The ORR was also significantly higher in the nivolumab arm compared with everolimus (25% vs 5%; *P* < 0.001). Median PFS was 4.6 months with nivolumab and 4.4 months with everolimus (*P* = 0.11). The most common treatment-related AEs in patients treated with nivolumab monotherapy were fatigue (33%), nausea (14%), and pruritus (14%) [7]. On the basis of these results, nivolumab is approved in Europe and the United States for treatment of patients with aRCC who have received prior therapy [7, 21, 22]. Nivolumab has further demonstrated clinical benefit in combination with ipilimumab (a monoclonal antibody that blocks cytotoxic T-lymphocyte–associated antigen-4 immune checkpoint signaling) in previously treated and treatment-naïve patients with aRCC, and this combination is now approved for the treatment of patients with intermediate- or poor-risk, treatment-naïve aRCC in the United States [21, 23, 24].

The antitumor activity of VEGF TKIs is attributed to their effect on angiogenesis, however, emerging data suggest that these agents may exert positive immune-modulatory activity in the suppressive tumor immune microenvironment. For example, sunitinib reduces the accumulation of myeloid-derived suppressor cells and reverses suppression of T cells in patients with aRCC [25, 26]. The combination of immune checkpoint inhibitors plus TKI agents warrants further investigation. Other clinical trials have investigated the efficacy and safety of combination TKI and checkpoint inhibitor–based therapy in patients with aRCC [27, 28]. Preliminary results from these studies have shown clinical benefit, however, some combinations have resulted in unacceptable toxicity [29].

Here, we report 3-year outcomes from the open-label, parallel-cohort, dose-escalation, phase I CheckMate 016 study of patients with aRCC treated with a combination of nivolumab and the TKIs sunitinib or pazopanib.

## Methods

### Study design

CheckMate 016 was a multicenter, open-label, phase I study. We report here the safety and efficacy outcomes of patients assigned to either nivolumab plus sunitinib (arm N + S) or nivolumab plus pazopanib (arm N + P). The safety and efficacy outcomes for CheckMate 016 patients assigned to different nivolumab plus ipilimumab treatment regimens have been reported previously [23]. Patients were assigned to treatment arms N + S and N + P in two phases: an escalation phase to determine the maximum tolerated dose (MTD) to gain safety and tolerability information, and a planned expansion phase to gain additional safety information.

### Dosing

The starting dose of nivolumab was 2 mg/kg of body weight intravenously every 3 weeks (N2; dose-escalation phase), with planned increase to 5 mg/kg intravenously every 3 weeks (N5; dose-expansion phase). Each treatment cycle was 6 weeks in duration; patients received nivolumab on days 1 and 22 in combination with sunitinib (50 mg orally on days 1–28 of each 6-week cycle; arm N + S) or pazopanib (800 mg orally on each day of the 6-week cycle; arm N + P) until disease progression/unacceptable toxicity. Expansion phase recruitment was dependent on the MTD assessed by the modified toxicity probability interval [30] during dose escalation. If the MTD of nivolumab was ≥5 mg/kg in either arm, the arm was further expanded to include treatment-naïve patients. Patients could discontinue treatment due to investigator-assessed, Response Evaluation Criteria in Solid Tumors (RECIST) v1.1-defined disease progression, unacceptable toxicity, withdrawal of consent, or per the investigator’s clinical judgment. If the combined incidence of treatment-related toxicity required discontinuation of >30% of treated patients, further enrollment to that arm was paused and a decision on whether to continue dosing was made based on the observed aggregate (acute and chronic) toxicities.

Sunitinib and pazopanib dose delays, reductions, and escalations were permitted per the approved product labels. All dose reductions of sunitinib were in 12.5-mg increments and were relative to the lowest dose level of the current cycle. The initial intra-patient dose reduction of pazopanib was to 400 mg. Additional pazopanib dose reductions were in 200-mg increments and were relative to the lowest dose level of the current cycle. If the current dose level was 25 mg (sunitinib) or 200 mg (pazopanib) and the toxicity guidelines required a further permanent dose reduction to mitigate sunitinib or pazopanib-related toxicity, the patient was discontinued from receiving that study drug. The pazopanib or sunitinib dosing period could not be extended to compensate for interruptions in study treatment. Nivolumab intra-patient dose reductions or escalations were not permitted, however, administration could be delayed based on specific AE criteria. Patients could resume treatment with nivolumab, pazopanib, or sunitinib when treatment-related AE(s) resolved to grade 1 or baseline. If a treatment-related AE occurred after cycle 1 and met criteria for discontinuation but was attributable to the TKI and not to nivolumab, or if a patient stopped the TKI secondary to chronic toxicity, continuation on nivolumab monotherapy was permitted.

### Patients

Patients eligible for inclusion were ≥18 years of age with histologically confirmed aRCC with a clear cell component (escalation and expansion phases) or non–clear cell RCC, limited to papillary, chromophobe or unclassified histology (escalation phase only), had measurable disease per RECIST v1.1 criteria, Karnofsky performance status ≥80%, and were categorized with favorable- or intermediate-risk Memorial Sloan Kettering Cancer Center prognostic score at study enrollment. Patients were required to have received ≥1 prior systemic treatment regimen in the advanced/metastatic setting to be eligible for the escalation phase. Patients eligible for the treatment-naïve expansion phase were not permitted to have received any prior systemic therapy in the advanced/metastatic setting. Patients who received prior pazopanib were assigned to arm N + S, while those who received prior sunitinib were assigned to arm N + P. Patients with prior treatment other than sunitinib or pazopanib could be assigned to either arm. Patients who received prior sunitinib or pazopanib and previously required permanent discontinuation due to toxicity, or required dose reduction/delay during the first 12 weeks of therapy due to toxicity were excluded, as were patients who had received both prior sunitinib and pazopanib. Patients with active central nervous system metastases, poorly controlled hypertension, evidence of active bleeding or bleeding susceptibility within 30 days of enrollment, or impairment of gastrointestinal function or gastrointestinal disease that may have significantly altered the absorption of either antiangiogenic TKI were excluded. Patients with current or recent history of a known or suspected autoimmune disorder requiring systemic corticosteroids equivalent to ≥10 mg of oral prednisone were also excluded.

### Study endpoints and assessments

The primary objective was to assess overall safety and tolerability of nivolumab plus sunitinib or pazopanib in order to determine the MTD of these combination regimens. Safety and tolerability were defined by incidence of AEs occurring ≤100 days after the last study treatment dose, and the worst toxicity grade of clinical laboratory tests, including hematology, comprehensive metabolic profile, and urinalysis. AEs were graded according to National Cancer Institute Common Terminology Criteria for Adverse Events v4.0. Additional safety assessments included determination of treatment-related AEs leading to discontinuation and any-grade select treatment-related AEs, defined as those with possible immune-mediated etiology.

Secondary endpoints included ORR, duration of response (DoR), and PFS, all investigator-assessed per RECIST v1.1. ORR was defined as the proportion of all treated patients whose best overall response was a complete or a partial response. DoR was calculated for all treated patients who achieved a complete or partial response, with DoR defined as the time between dates of first response and of disease progression or death, whichever occurred first. PFS was defined as the time from dates of first study medication dose to first disease progression or death. OS, an exploratory endpoint, was defined as the time from date of first dose of study medication to the date of death (any reason). If the patient did not die, OS was censored on the last date the subject was known to be alive. PFS and OS rates were calculated over time. Tumor assessments were done at screening, every 6 weeks (±1 week) from the first study treatment dose for the first four patient visits, and every 12 weeks (±1 week) thereafter until disease progression.

### Statistical analysis

The study sample size required to determine MTD in this phase I dose-escalation trial for each dose was dependent on observed toxicity and posterior inference. Six eligible patients per arm were to be treated with the N2 dosing regimen initially. Additional patients could be assigned to either the same or the higher nivolumab dose level cohort based on the number of dose-limiting toxicities (DLTs) observed. Depending on the number observed, de-escalation could occur without the possibility of re-escalation. If deemed safe, additional patients were to be treated at the N5 level in combination with sunitinib or pazopanib to gain additional safety information. Administration of N5 to 26 or 32 patients was determined adequate to provide 90% probability of observing ≥1 occurrence of any AE that would occur with an 8% or 7% incidence in the population from which the study sample was selected for the N + S or N + P arms, respectively. At the end of the trial, the MTD was estimated as the dose with the smallest difference of estimated and target toxicity across all doses.

Safety and efficacy analyses included all patients who received ≥1 dose of study medication in either arm. AEs were summarized and reported by organ system, preferred term, treatment arm, and dose cohort, coded per MedDRA. ORR and its 95% exact confidence interval (CI) were determined by Clopper and Pearson methodology, while the Kaplan–Meier method was used to analyze DoR and its 95% CI. PFS and OS were plotted using the Kaplan–Meier method, with median and corresponding two-sided 95% CIs reported. PFS and OS rate point estimates were derived from Kaplan–Meier analyses. Statistical analyses comparing safety between arms were not performed.

## Results

### Patient population and baseline characteristics

A total of 194 patients were enrolled in the study from February 2012 to May 2014; 153 were treated, with 33 assigned to arm N + S and 20 assigned to arm N + P (Additional file 1: Table S1). The remainder received nivolumab plus ipilimumab as previously reported [23]. In arm N + S, seven patients completed the dose-escalation phase at the N2 dose, with a further 26 patients included in the dose-expansion phase at the N5 dose (*N* = 33). In arm N + S, 18 (55%) patients had one or more dose reductions of sunitinib and 21 (64%) patients had at least one nivolumab dose delay. In arm N + P, seven (35%) patients had one or more dose reductions of pazopanib and 11 (55%) patients had at least one nivolumab dose delay. Arm N + P was not expanded beyond the N2 dose as per prespecified criteria for DLTs; three patients had elevated ALT/AST and one had fatigue. Fourteen (42.4%) patients in arm N + S had received ≥1 prior systemic therapy, and 19 (57.6%) patients (all enrolled in the N + S expansion arm) were treatment-naïve. All 20 patients in arm N + P had received ≥1 prior systemic therapy.

Baseline demographic and clinical characteristics are detailed in Table 1. At data cutoff (June 12, 2017), median follow-up was 50.0 (N + S) and 27.1 (N + P) months. Median duration of therapy was 45.1 weeks for nivolumab and 28 weeks for sunitinib (N + S); median duration of therapy was 15.1 weeks for nivolumab and 13.9 weeks for pazopanib (N + P).

**Table 1:** Baseline demographic and clinical characteristics of treated patients

Characteristic	N + S (*N* = 33)	N + P (*N* = 20)
Age, years		
Median (range)	57.0 (38–75)	56.0 (40–72)
Age <65 years, *n* (%)	24 (72.7)	17 (85.0)
Sex, *n* (%)		
Male	26 (78.8)	18 (90.0)
Female	7 (21.2)	2 (10.0)
Race, *n* (%)		
Caucasian	28 (84.8)	18 (90.0)
Asian	1 (3.0)	0
Black/African American	2 (6.1)	1 (5.0)
Other	2 (6.1)	1 (5.0)
Ethnicity, *n* (%)		
Hispanic/Latino	2 (6.1)	0
Not Hispanic/Latino	29 (87.9)	18 (90.0)
Not reported	2 (6.1)	2 (10.0)
MSKCC risk category, *n* (%)		
Favorable	16 (48.5)	4 (20.0)
Intermediate	16 (48.5)	14 (70.0)
Poor	1 (3.0)	2 (10.0)
Prior surgery, *n* (%)	33 (100.0)	20 (100.0)
Prior radiotherapy, *n* (%)	5 (15.2)	10 (50.0)
Prior systemic therapy, *n* (%)	14 (42.4)	20 (100.0)
VEGFR inhibitor	5 (15.2)	17 (85.0)
Other antiangiogenic	7 (21.2)	17 (85.0)
Cytokine	9 (27.3)	10 (50.0)
mTOR inhibitor	0	3 (15.0)
Other	3 (9.1)	4 (20.0)
No. of prior therapies, *n* (%)		
0	19 (57.6)	0
1	14 (42.4)	14 (70.0)
2	0	4 (20.0)
3	0	1 (5.0)
≥4	0	1 (5.0)
Treatment setting, ^a^ *n* (%)		
Adjuvant	3 (9.1)	4 (20.0)
Metastatic	0	2 (10.0)
Neoadjuvant	11 (33.3)	16 (80.0)

^a^More than one setting per patient may be reflected in the frequency

### Safety and tolerability

Among all patients assigned to either arms N + S or N + P, 100% experienced a treatment-related AE of any grade, and 81.8% and 70.0% experienced a grade 3 or 4 treatment-related AE, respectively (Table 2). There were no grade 5 treatment-related AEs in either study arm. Select treatment-related AEs (those with possible immune-mediated etiology) included skin, endocrine, gastrointestinal, hepatic, renal, and pulmonary events (Table 2).

**Table 2:** TRAEs (in ≥30% of patients), select TRAEs, and TRAEs leading to discontinuation in ≥2 patients

TRAE, preferred term, *n* (%)^a^	Treatment arm			
	N + S (*N* = 33)		N + P (*N* = 20)	
	All grades	Grade 3 or 4	All grades	Grade 3 or 4
Total patients with an event	33 (100.0)	27 (81.8)	20 (100.0)	14 (70.0)
Fatigue	28 (84.8)	3 (9.1)	12 (60.0)	3 (15.0)
Diarrhea	21 (63.6)	3 (9.1)	12 (60.0)	4 (20.0)
Dysgeusia	21 (63.6)	0	10 (50.0)	0
Nausea	19 (57.6)	1 (3.0)	15 (75.0)	0
Hypertension	16 (48.5)	6 (18.2)	5 (25.0)	2 (10.0)
Decreased appetite	16 (48.5)	1 (3.0)	8 (40.0)	0
Increased ALT	13 (39.4)	6 (18.2)	5 (25.0)	4 (20.0)
Palmar-plantar erythrodysesthesia syndrome	13 (39.4)	0	0	0
Increased AST	12 (36.4)	3 (9.1)	6 (30.0)	4 (20.0)
Blood creatinine increased	11 (33.3)	2 (6.1)	1 (5.0)	0
Hypothyroidism	11 (33.3)	0	4 (20.0)	1 (5.0)
Dyspepsia	11 (33.3)	0	4 (20.0)	0
Dry skin	11 (33.3)	0	2 (10.0)	0
Mucosal inflammation	10 (30.3)	0	5 (25.0)	0
Dry mouth	10 (30.3)	0	1 (5.0)	0
Arthralgia	8 (24.2)	0	7 (35.0)	1 (5.0)
Pruritus	8 (24.2)	0	7 (35.0)	0
Vomiting	7 (21.2)	1 (3.0)	6 (30.0)	0
Select TRAE, organ class, *n* (%)^b^				
Skin	26 (78.8)	2 (6.1)	11 (55.0)	0
Endocrine	12 (36.4)	0	5 (25.0)	2 (10.0)
Gastrointestinal	21 (63.6)	3 (9.1)	12 (60.0)	4 (20.0)
Hepatic	15 (45.5)	8 (24.2)	7 (35.0)	4 (20.0)
Renal	13 (39.4)	4 (12.1)	1 (5.0)	0
Pulmonary	1 (3.0)	1 (3.0)	1 (5.0)	0
TRAE leading to discontinuation, preferred term, *n* (%)^a^				
Total patients with an event	13 (39.4)	11 (33.3)	5 (25.0)	4 (20.0)
Increased ALT	3 (9.1)	2 (6.1)	3 (15.0)	3 (15.0)
Acute kidney injury	3 (9.1)	1 (3.0)	0	0
Increased AST	1 (3.0)	1 (3.0)	3 (15.0)	3 (15.0)

^a^Includes events reported between the first dose and 100 days after the last dose of study therapy

^b^Includes events reported between the first dose and 30 days after the last dose of study therapy

For patients in arm N + S, the most common any-grade treatment-related AEs were fatigue (84.8%). diarrhea (63.6%), dysgeusia (63.6%), and nausea (57.6%). The most common grade 3 or 4 treatment-related AEs were hypertension (18.2%), increased ALT (18.2%), increased AST (9.1%), diarrhea (9.1%), and fatigue (9.1%). Treatment-related AEs of any grade leading to discontinuation occurred in 13 (39.4%) patients in this arm (Table 2), and 13 (39.4%) patients received a systemic corticosteroid to manage AEs (Additional file 2: Figure S1).

For patients in arm N + P, the most common any-grade treatment-related AEs were also fatigue (60.0%), diarrhea (60.0%), dysgeusia (50.0%), and nausea (75.0%). Similarly, the most common grade 3 or 4 treatment-related AEs were hypertension (10.0%), increased ALT (20.0%), increased AST (20.0%), diarrhea (20.0%), and fatigue (15.0%). Treatment-related AEs of any grade leading to discontinuation occurred in five (25.0%) patients in this arm (Table 2), and 12 (60.0%) patients received a systemic corticosteroid to manage AEs (Additional file 2: Figure S1).

### Efficacy

**Fig. 1:**
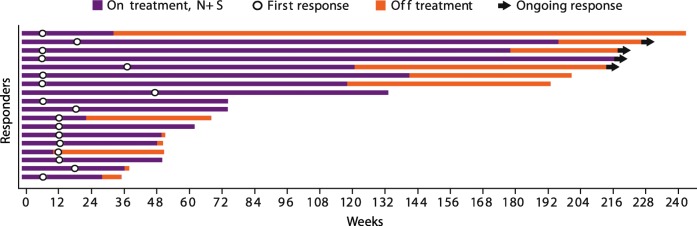
Time to response, duration of response, and time on therapy (weeks) in arm N + S. Patients with confirmed response are presented (*n* = 18)

**Fig. 2:**
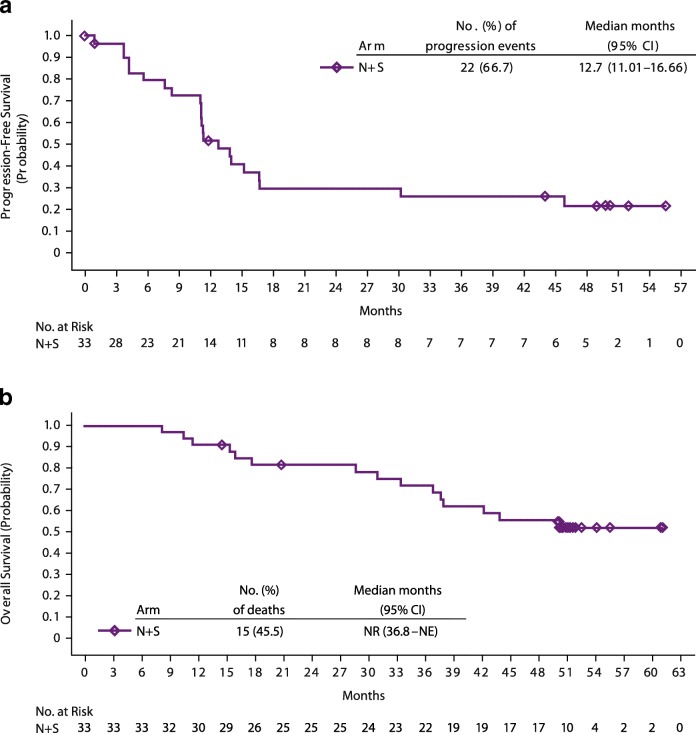
Kaplan–Meier plots of progression-free survival (**a**) and overall survival (**b**) in arm N + S

In treatment arm N + S, the confirmed ORR (95% CI) was 54.5% (36.4–71.9). Two (6.1%) patients achieved a complete response, 16 (48.5%) achieved a partial response, 11 (33.3%) had stable disease, one (0.3%) had progressive disease, and in three patients (9.1%), response was undeterminable. Responses were sustained with a median (95% CI) DoR of 60.2 (37.1–not reached [NR]) weeks. Four of the 18 responders (22.2%) in this arm have an ongoing response as of the data cutoff (Fig. 1); notably, eight of the 18 responders (44.4%) had a response that was sustained for ≥6 months after discontinuation of therapy, with one responder maintaining a response for more than 4 years after discontinuing N + S therapy. Most patients with a baseline and ≥ 1 post-baseline assessment experienced a reduction in target lesion size; 20 of 30 evaluable patients in this arm experienced a reduction of ≥30% (Additional file 3: Figure S2). Median (95% CI) PFS was 12.7 (11.0–16.7) months (Fig. 2a). PFS rates at 6, 12, 18, and 24 months were 79.4%, 51.8%, 29.6%, and 29.6%. At a median follow-up of 50.0 months, the median OS was NR (36.8–NR) (Fig. 2b). OS rates at 12, 18, and 24 months were 90.9%, 81.5%, and 81.5%. Among treated patients, 45.5% in this arm received subsequent medical intervention, with 42.4% receiving systemic therapy.

**Fig. 3:**
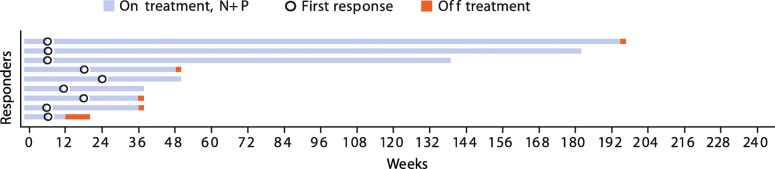
Time to response, duration of response, and time on therapy (weeks) in arm N + P. Patients with confirmed response are presented (*n* = 9, no ongoing responses were observed)

**Fig. 4:**
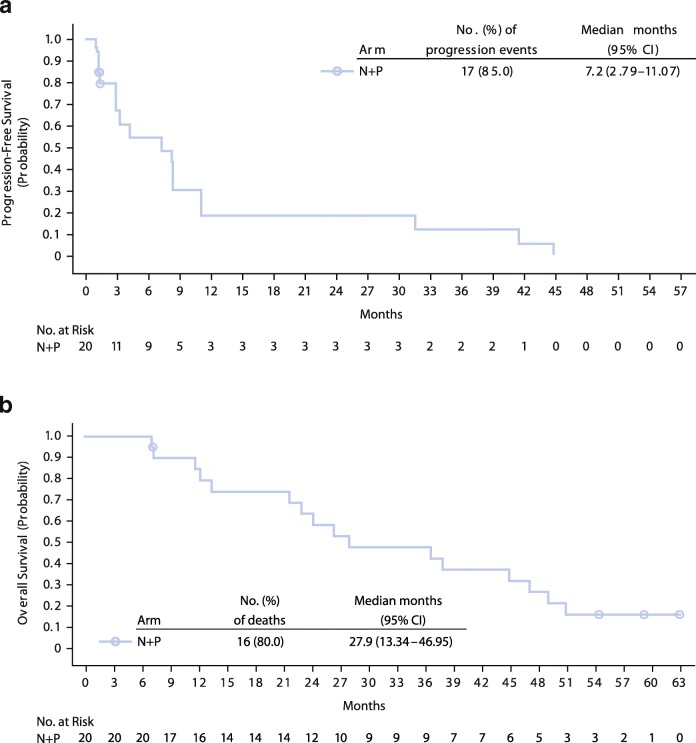
Kaplan–Meier plots of progression-free survival (**a**) and overall survival (**b**) in arm N + P

In treatment arm N + P, the confirmed ORR (95% CI) was 45.0% (23.1–68.5). There were no complete responses, nine (45.0%) patients had partial responses, seven (35.0%) had stable disease, and four (20.0%) had progressive disease. Responses were sustained with a median (95% CI) DoR of 30.1 (12.1–174.1) weeks (Fig. 3). Ten of 19 evaluable patients treated with N + P experienced a reduction in target lesion size of ≥30% (Additional file 4: Figure S3). Median (95% CI) PFS was 7.2 (2.8–11.1) months (Fig. 4a). The 6-month PFS rate was 54.9%, and not calculated for the subsequent months in this arm. At a median follow-up of 27.1 months, median OS (95% CI) was 27.9 months (13.3–47.0) (Fig. 4b). OS rates at 12, 18, and 24 months were 84.4%, 73.9%, and 63.3%. Among treated patients, 80.0% received subsequent medical intervention, with 70.0% receiving systemic therapy.

## Discussion

In this first study to combine nivolumab with antiangiogenic TKIs, notable clinical activity was observed in patients with aRCC, albeit with substantial toxicity. Extended follow-up of the CheckMate 016 study in aRCC did not reveal any late-emergent select AEs with the nivolumab plus TKI combinations [31]. However, both N + S and N + P combinations resulted in greater frequencies of high-grade/treatment-related AEs and AEs leading to discontinuation than previously observed with nivolumab, sunitinib, or pazopanib monotherapy.

Based on the safety results in the N2 dose-escalation phase, arm N + S advanced to expansion at the N5 dose level, while arm N + P was closed due to early DLTs observed in the initial escalation phase. Overall, 81.8% and 70.0% of patients in arms N + S and N + P, respectively, experienced a treatment-related grade 3 or 4 AE. In comparison, a similar proportion of patients previously experienced grade 3 or 4 treatment-related AEs with sunitinib (69% of patients treated for 0–4 years, data pooled from 807 patients across multiple trials) [32]. However, this rate was higher than the rate of all-cause grade 3 or 4 AEs previously reported with pazopanib monotherapy (33% or 7% of patients, respectively, in a phase III study [*N* = 435]) [11], and higher than the rate of grade 3 or 4 treatment-related AEs previously reported with nivolumab monotherapy (19% of patients in a phase III study [*N* = 410]) [7]. Any-grade and grade 3 or 4 treatment-related select AEs also occurred more frequently in patients treated with N + S and N + P versus those treated in the nivolumab 3 mg/kg plus ipilimumab 1 mg/kg (*N* = 47) arm of the CheckMate 016 trial reported earlier [23].

Treatment-related AEs of any grade leading to discontinuation occurred in 39.4% and 25.0% of patients in arms N + S and N + P. In comparison, previous trials have reported that 11% of patients with cytokine-refractory aRCC discontinued sunitinib treatment due to all-cause AEs [10]; 19% (pretreated) and 12% (treatment-naïve) of patients discontinued pazopanib treatment due to all-cause AEs [11]; and 8% of patients with aRCC who received second-line nivolumab monotherapy discontinued due to a treatment-related AE [7]. In the phase III trial of first-line nivolumab plus ipilimumab versus sunitinib, 22% of patients in the nivolumab plus ipilimumab combination arm and 12% in the sunitinib arm reported treatment-related AEs leading to discontinuation [24]. An important outcome of the current study, and one that was observed in the nivolumab plus ipilimumab arm of CheckMate 016 [23], as well as with other immune checkpoint inhibitor-based regimens in various tumor types [33–36], is that responses were noted to continue beyond treatment discontinuation.

While associated with substantial toxicity, the addition of sunitinib or pazopanib to nivolumab showed sustained antitumor activity in this small, favorable or intermediate risk, mixed population of treatment-naïve and pretreated aRCC patients, with more durable responses compared with monotherapy. Confirmed ORRs of 54.5% and 45.0% were reported in arms N + S and N + P, respectively, with median DoRs of 60.2 and 30.1 weeks. High ORRs have also been reported with other immune checkpoint inhibitor plus antiangiogenic combinations in early-phase studies of aRCC. These include pembrolizumab in combination with axitinib (ORR 73% in 52 treatment-naïve patients) [27] or lenvatinib (ORR 63.3% in 30 treatment-naïve and pretreated patients) [28]; avelumab in combination with axitinib (ORR 58% in 55 treatment-naïve RCC patients) [37]; and atezolizumab in combination with bevacizumab (ORRs of 32% in 101 treatment-naïve RCC patients [phase II] [38] and 37% in 454 treatment-naïve RCC patients [phase III]) [39]. Safety results from the aforementioned combination studies were reported as acceptable and in most cases comparable to previous reports of either agent as monotherapy [27, 28, 37–39]. A previous phase I/II study assessing the safety and efficacy of pembrolizumab in combination with pazopanib in patients with aRCC demonstrated preliminary efficacy albeit with significant hepatotoxicity [29], suggesting that the choice of TKI may impact the overall risk versus benefit outcome of the various combination therapies under investigation.

In the current study, which at present has the longest follow-up for a combination regimen based on an immune checkpoint inhibitor and a TKI, favorable antitumor activity and survival benefits were observed in arm N + S. Two (6.1%) patients had a complete response and 16 (48.5%) achieved partial response. Most responses occurred shortly after treatment initiation, and were of notable magnitude in both arms. Of all patients in arm N + S who had a baseline target lesion and at least one post-baseline assessment, zero patients had increases in target lesion tumor burden, and 67% of patients had a reduction of ≥30% in their target lesion tumor burden at a median follow-up of >4 years. Median OS was NR at the time of this analysis in arm N + S, and was 27.9 months in arm N + P. The longer median duration of nivolumab treatment in arm N + S (45.1 weeks) versus arm N + P (15.1 weeks) may be attributable to inclusion of treatment-naïve patients and a longer duration of benefit in this arm.

## Conclusions

While the duration and depth of response observed in arm N + S was notable, the toxicity observed in this study with the currently approved standard dose of sunitinib or pazopanib in combination with nivolumab precludes further clinical evaluation of either combination. The tolerability results observed in the current study, particularly in arm N + P, may reflect toxicity due to the choice and standard dose of the TKI rather than nivolumab toxicity. Indeed, as mentioned previously, the combination of pembrolizumab and pazopanib (at the same dose as used in this study) was associated with significant hepatotoxicity [29], but regimens comprising pembrolizumab and axitinib or lenvatinib appear to be associated with more manageable safety profiles [27, 28]. This suggests that the respective efficacy and safety of combination regimens based on immune checkpoint inhibitors and antiangiogenic drugs may depend on selection of the antiangiogenic component. Nevertheless, confidence in the concept of combined immune checkpoint blockade and antiangiogenesis is demonstrated by the number of ongoing phase III studies evaluating the combination of an immune checkpoint inhibitor with an anti-VEGF TKI [39–43]. These ongoing studies will help further define the role of these combinations in the evolving armamentarium for treating aRCC.

### Limitations

This small phase I study sought to determine a safe and tolerable dose of nivolumab as part of a combination regimen with standard doses of the TKIs sunitinib or pazopanib, to enable further evaluation of the safety and efficacy of such combinations in patients with aRCC. This study was only powered to assess overall safety and tolerability in order to determine the MTD and recommended phase II dose of each combination regimen in this setting. The antitumor activity of nivolumab plus TKI combinations was assessed as a secondary endpoint in this study by the investigator-assessed RECIST v1.1 criteria. Additionally, due to the DLTs observed preventing the expansion of arm N + P, this arm contained only pretreated patients, while the N + S arm contained a mixed population of pretreated patients (nivolumab 2 mg/kg plus sunitinib 50 mg) and treatment-naïve patients (nivolumab 5 mg/kg plus sunitinib 50 mg). No direct comparisons can therefore be made regarding relative efficacy or safety between nivolumab plus sunitinib or nivolumab plus pazopanib combination regimens, or between either combination therapy and any monotherapy. Ongoing studies will help determine if different dosing regimens, or different immuno-oncology plus TKI combinations, could yield safe and efficacious outcomes for patients with aRCC.

## Additional files

Additional file 1: **Table S1.** Concomitant systemic corticosteroids for adverse event management. (DOCX 46 kb)

Additional file 2: **Figure S1.** Patient disposition. (DOCX 512 kb)

Additional file 3: **Figure S2.** Best percent change from baseline in target lesion tumor burden up to Response Evaluation Criteria in Solid Tumors version 1.1 (RECIST v1.1) progression. Dashed lines denote 30% decrease and 20% increase in tumor burden. Patients whose target lesion resolved 100% may have had concurrent progression of nontarget lesions. Patients with baseline target lesion and at least one post-baseline assessment of target lesion are presented (N+S, *n* = 30). (DOCX 229 kb)

Additional file 4: **Figure S3.** Best percent change from baseline in target lesion tumor burden up to Response Evaluation Criteria in Solid Tumors version 1.1 (RECIST v1.1) progression. Dashed lines denote 30% decrease and 20% increase in tumor burden. Patients whose target lesion resolved 100% may have had concurrent progression of nontarget lesions. Patients with baseline target lesion and at least one post-baseline assessment of target lesion are presented (N+P, *n* = 19). (DOCX 177 kb)

## Abbreviations

AE: adverse event
ALT: alanine aminotransferase
aRCC: advanced or metastatic renal cell carcinoma
AST: aspartate aminotransferase
CI: confidence interval
DLT: dose-limiting toxicity
DoR: duration of response
MSKCC: Memorial Sloan Kettering Cancer Center
MTD: maximum tolerated dose
mTOR: mammalian target of rapamycin
N + P: nivolumab 2 mg/kg plus pazopanib 800 mg
N + S: nivolumab 2 mg/kg or 5 mg/kg plus sunitinib 50 mg
N2: nivolumab 2 mg/kg
N5: nivolumab 5 mg/kg
NR: not reached
ORR: objective response rate
OS: overall survival
PD-1: programmed death-1
PFS: progression-free survival
RECIST: Response Evaluation Criteria in Solid Tumors
SD: standard deviation
Select TRAEs: treatment-related adverse events with possible immune-mediated etiology
TRAEs: treatment-related adverse events
TKI: tyrosine kinase inhibitor
VEGF: vascular endothelial growth factor
