# Correction to: Safety and efficacy of nivolumab in combination with sunitinib or pazopanib in advanced or metastatic renal cell carcinoma: the CheckMate 016 study

**DOI:** 10.1186/s40425-019-0559-3

**Published:** 2019-03-14

**Authors:** Asim Amin, Elizabeth R. Plimack, Marc S. Ernstoff, Lionel D. Lewis, Todd M. Bauer, David F. McDermott, Michael Carducci, Christian Kollmannsberger, Brian I. Rini, Daniel Y. C. Heng, Jennifer Knox, Martin H. Voss, Jennifer Spratlin, Elmer Berghorn, Lingfeng Yang, Hans J. Hammers

**Affiliations:** 1 Immunotherapy program, Levine Cancer Institute, Carolinas HealthCare System, 1024 Morehead Medical Drive, Charlotte, NC 28204 USA; 2 Division of Genitourinary Medical Oncology, Department of Hematology/Oncology, Fox Chase Cancer Center, Philadelphia, PA 19111 USA; 3 Division of Oncology, Department of Medicine, Roswell Park Cancer Institute, Elm and Carlton Streets, Buffalo, NY 14203 USA; 4 Department of Medicine at The Geisel School of Medicine and The Norris Cotton Cancer Center at Dartmouth-Hitchcock Medical Center, Lebanon, NH 03756 USA; 5 Sarah Cannon Research Institute/Tennessee Oncology, PLLC, Nashville, TN 37203 USA; 6 Department of Medicine, Beth Israel Deaconess Medical Center, Dana-Farber/Harvard Cancer Center, Boston, MA 02215 USA; 7 Department of Oncology, Johns Hopkins Sidney Kimmel Comprehensive Cancer Center, Baltimore, MD 21287 USA; 8 Division of Medical Oncology, British Columbia Cancer Agency, Vancouver, BC V5Z 4E6 Canada; 9 Lerner College of Medicine, Department of Hematology and Oncology, Cleveland Clinic Taussig Cancer Institute, Cleveland, OH 44195 USA; 10 Department of Oncology, Tom Baker Cancer Center, University of Calgary, Calgary, AB T2N 4N2 Canada; 11 Cancer Clinical Research Unit (CCRU), Princess Margaret Cancer Centre, Toronto, ON M5G 1Z5 Canada; 12 Department of Medicine, Memorial Sloan Kettering Cancer Center, New York, NY 10065 USA; 13 Department of Oncology, Cross Cancer Institute, University of Alberta, Edmonton, AB T6G 1Z2 Canada; 14 Oncology - Global Clinical Research, Bristol-Myers Squibb, Princeton, NJ 08541 USA; 15 Department of Internal Medicine, UT Southwestern – Kidney Cancer Program, Dallas, TX 75390 USA

**Correction to: Journal for ImmunoTherapy of Cancer (2018) 6:109**

**https://doi.org/10.1186/s40425-018-0420-0**

Following publication of the original article [1], the author reported a mistake related to a percentage. In page 5 the sentence “Two (6.1%) patients achieved a complete response, 16 (48.5%) achieved a partial response, 11 (33.3%) had stable disease, one (0.3%) had progressive disease, and in three patients (9.1%), response was undeterminable.” should be replaced with “Two (6.1%) patients achieved a complete response, 16 (48.5%) achieved a partial response, 11 (33.3%) had stable disease, one (3.0%) had progressive disease, and in three patients (9.1%), response was undeterminable.” (3.0% instead of 0.3%).

The publisher apologizes for any inconvenience caused by this error.
