# Supplementary material for: Safety and efficacy of nivolumab in combination with sunitinib or pazopanib in advanced or metastatic renal cell carcinoma: the CheckMate 016 study
Source: J Immunother Cancer. 2018 Oct 22;6:109. doi: 10.1186/s40425-018-0420-0 (PMC6196426; doi:10.1186/s40425-018-0420-0)
Supplement: Supplementary file 1 — Table S1. Concomitant systemic corticosteroids for adverse event management. (DOCX 46 kb) [file 40425_2018_420_MOESM1_ESM.docx]

**Additional file 1: Table S1.** Concomitant systemic corticosteroids for adverse event management

| *n* (%) | N+S (*N* = 33) | N+P (*N* = 20) |
| --- | --- | --- |
| Corticosteroid, systemic  Prednisone  Corticosteroid  Dexamethasone  Methylprednisolone | 13 (39.4)  11 (33.3)  1 (3.0)  1 (3.0)  7 (21.2) | 12 (60.0)  11 (55.0)  0  2 (10.0)  2 (10.0) |
