# Supplementary material for: Safety and efficacy of nivolumab in combination with sunitinib or pazopanib in advanced or metastatic renal cell carcinoma: the CheckMate 016 study
Source: J Immunother Cancer. 2018 Oct 22;6:109. doi: 10.1186/s40425-018-0420-0 (PMC6196426; doi:10.1186/s40425-018-0420-0)
Supplement: Supplementary file 2 — Figure S1. Patient disposition. (DOCX 512 kb) [file 40425_2018_420_MOESM2_ESM.docx]

**Additional file 2: Figure S1.** Patient disposition.

^a^Data published previously [1]


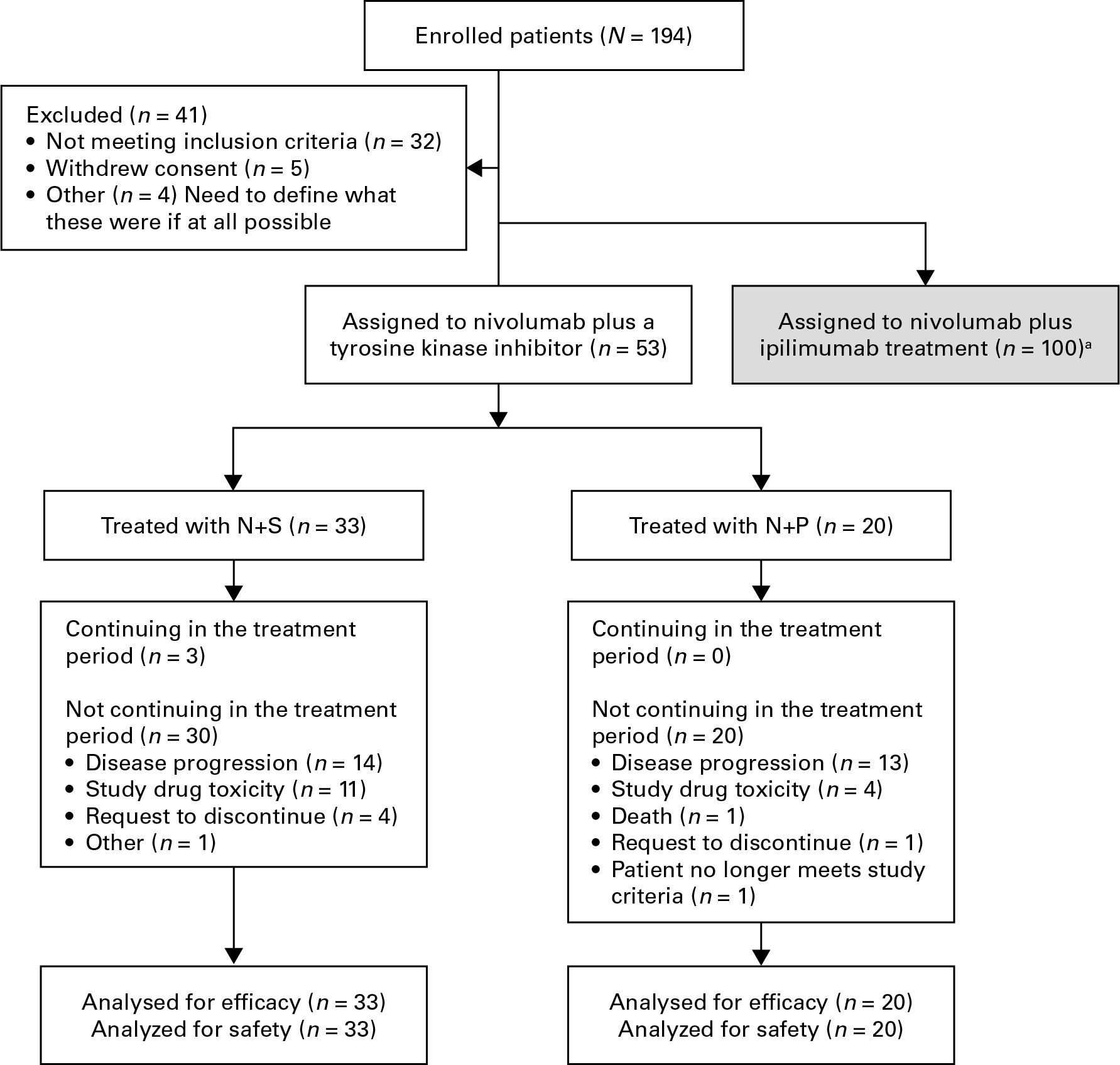


**Reference**

[1] Hammers HJ, Plimack ER, Infante JR, Rini BI, McDermott DF, Lewis LD, et al. Safety and efficacy of nivolumab in combination with ipilimumab in metastatic renal cell carcinoma: The CheckMate 016 study. *J Clin Oncol*. 2017;35(34):3851-8.
