# Supplementary material for: Safety and efficacy of nivolumab in combination with sunitinib or pazopanib in advanced or metastatic renal cell carcinoma: the CheckMate 016 study
Source: J Immunother Cancer. 2018 Oct 22;6:109. doi: 10.1186/s40425-018-0420-0 (PMC6196426; doi:10.1186/s40425-018-0420-0)

**Additional file 3: Figure S2** Best percent change from baseline in target lesion tumor burden up to Response Evaluation Criteria in Solid Tumors version 1.1 (RECIST v1.1) progression. Dashed lines denote 30% decrease and 20% increase in tumor burden. Patients whose target lesion resolved 100% may have had concurrent progression of nontarget lesions. Patients with baseline target lesion and at least one post-baseline assessment of target lesion are presented (N+S, *n* = 30).


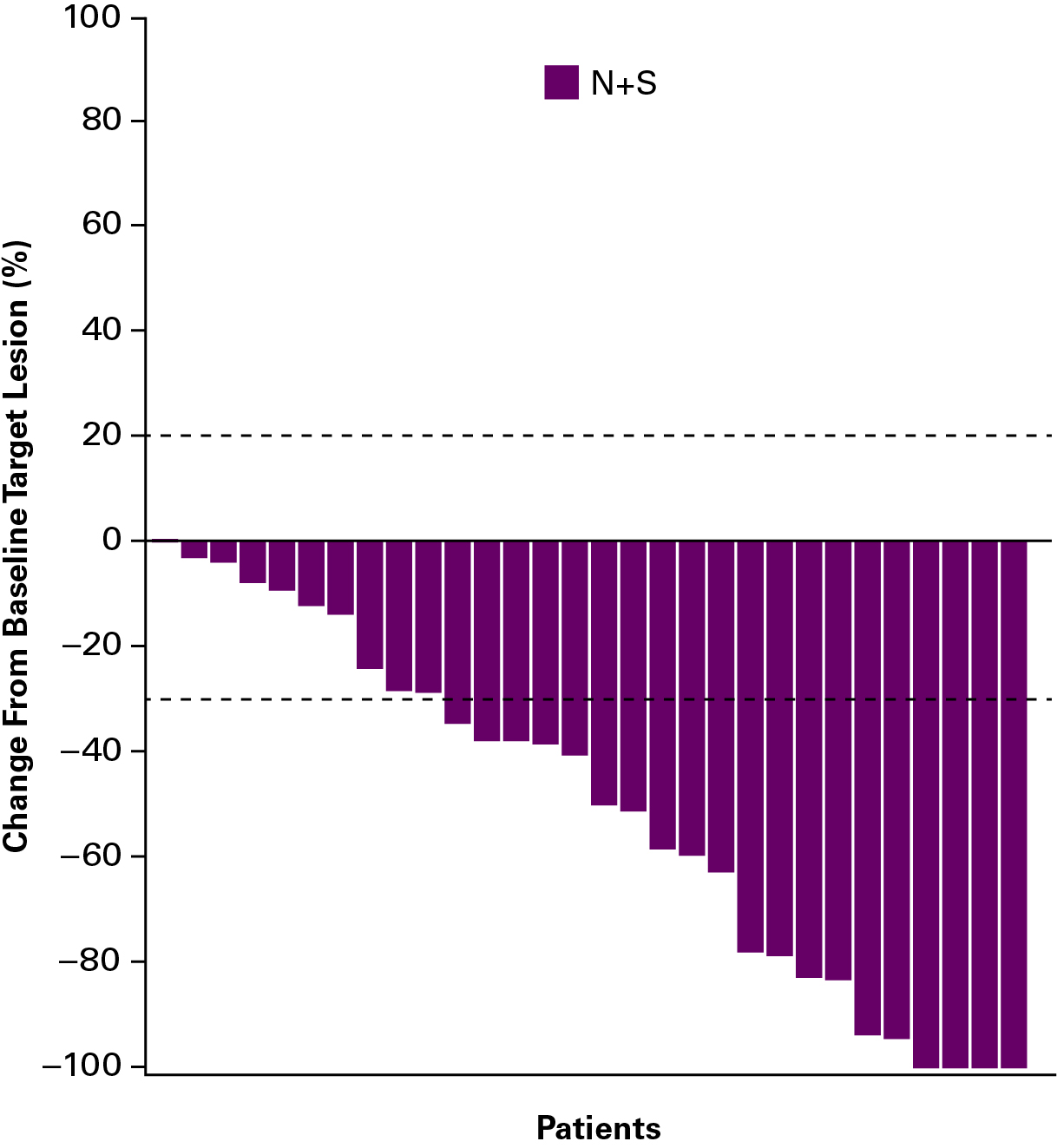

Supplement: Supplementary file 3 — Figure S2. Best percent change from baseline in target lesion tumor burden up to Response Evaluation Criteria in Solid Tumors version 1.1 (RECIST v1.1) progression. Dashed lines denote 30% decrease and 20% increase in tumor burden. Patients whose target lesion resolved 100% may have had concurrent progression of nontarget lesions. Patients with baseline target lesion and at least one post-baseline assessment of target lesion are presented (N+S, n = 30). (DOCX 229 kb) [file 40425_2018_420_MOESM3_ESM.docx]
